# Dietary Intake, Diet Quality, and Mealtime Experiences of Community-Dwelling Older Adults in The Netherlands: A Systematic Review

**DOI:** 10.3390/nu18213590

**Published:** 2026-10-29

**Authors:** Bart van der Wijst, Henriëtte S. Brummer, Rosalie van der Vaart, Astrid M. H. Horstman, Luc P. de Witte

**Affiliations:** 1 Technology for Health Care Research Group, The Hague University of Applied Sciences, Johanna Westerdijkplein 75, 2521 EN The Hague, The Netherlands; h.s.brummer@hhs.nl (H.S.B.); r.vandervaart@hhs.nl (R.v.d.V.); l.p.dewitte@hhs.nl (L.P.d.W.); 2 Nutrition and Dietetics, The Hague University of Applied Sciences, Johanna Westerdijkplein 75, 2521 EN The Hague, The Netherlands; 3 Centre of Expertise Health Innovation, The Hague University of Applied Sciences, Johanna Westerdijkplein 75, 2521 EN The Hague, The Netherlands; a.m.h.horstman@hhs.nl

**Keywords:** older adults, community-dwelling, dietary intake, diet quality, malnutrition, protein intake, vitamin D, healthy ageing, mealtime experiences, The Netherlands

## Abstract

Background/Objectives: Most older adults in The Netherlands prefer to age in place, which makes adequate nutrition central to preventing malnutrition and maintaining independence. Evidence specific to community-dwelling older adults (≥65 years) in The Netherlands is limited. This systematic review synthesises the evidence on their dietary intake, diet quality and mealtime experiences. Methods: PubMed, Scopus and EBSCOhost/CINAHL were searched with a SPIDER-guided strategy (September 2026) for studies with data collected from January 2015 onward. Two reviewers independently screened, extracted and appraised the studies, using Joanna Briggs Institute checklists, and findings were synthesised narratively. Results: Fifteen publications on thirteen independent study populations were included: thirteen quantitative studies on dietary intake and diet quality and two qualitative studies. Across studies, a substantial proportion of participants had protein intakes below recommendations, dietary vitamin D intake was consistently low and supplement use often fell short of the national guideline. Intake of fish, dairy and meat (alternatives) was frequently suboptimal, vegetable intake was mixed and fruit intake relatively adequate. Where assessed, overall diet quality was reasonable but suboptimal. The qualitative studies pointed to ingrained habits, limited awareness of protein needs and reluctance to change. Conclusions: Dietary intake of community-dwelling older adults in The Netherlands appears suboptimal, most notably for protein and vitamin D, while their own experiences of food and mealtimes remain poorly understood. Nutritional guidance may be more acceptable when it builds on existing habits through small adjustments. Contemporary, nationally representative intake data, including adults aged 80 years and older, and further qualitative research are needed.

## 1. Introduction

The Dutch population is ageing rapidly, and the proportion of adults aged 65 years and older will continue to increase in the coming decades, accompanied by substantial growth in the number of adults aged 80 years and older [1]. This demographic shift is expected to further strain a healthcare system already under pressure, with a rising demand for health and long-term care and growing workforce shortages [1]. At the same time, older adults are remaining in their homes for longer periods, driven by a strong preference to age in place rather than relocate [2]. As physical and cognitive capacities decline with advancing age, maintaining health becomes more important to sustain independent living.

Nutrition is a fundamental determinant of health in later life. Malnutrition occurs frequently in Dutch community-dwelling older adults: 8.5% of adults aged ≥65 years screen positive for undernutrition with the Short Nutritional Assessment Questionnaire 65+ (${\mathrm{SNAQ}}^{65+}$), rising to 19.3% among those aged ≥85 years and 15.8% among those receiving formal home care [3]. Malnutrition is defined as “a state resulting from lack of intake or uptake of nutrition that leads to altered body composition (decreased fat free mass) and body cell mass leading to diminished physical and mental function and impaired clinical outcome from disease” [4].

In older adults, malnutrition arises from multiple interacting factors, illustrated by the DoMAP model (Determinants of Malnutrition in Aged Persons) [5]. The DoMAP model identifies three central mechanisms (low intake, reduced bioavailability, and high nutritional requirements), each driven by medical, functional, psychological, social, and environmental determinants that interact with one another and thereby increase the risk of malnutrition. Malnutrition prevention is therefore key to sustain physical and mental function and to facilitate independent living.

Among the multiple determinants captured in DoMAP, (low) dietary intake represents one of the most directly modifiable factors and a primary entry point for preventive action. Effective prevention therefore depends on a clear understanding of what older adults actually consume and how well their intake aligns with current dietary guidelines. Dietary intake refers to the consumption of food products (e.g., bread, vegetables) as well as the intake of nutrients, including macronutrients, micronutrients, and fluids. Diet quality refers to the extent to which intake aligns with established dietary guidelines in The Netherlands. In this review, diet quality is operationalised primarily through adherence to Dutch dietary guidelines (subscores of the Dutch Healthy Diet-index Food Frequency Questionnaire, DHD-FFQ) and nutrient adequacy, rather than as a fully holistic assessment of overall diet.

There is some (limited) information on dietary intake of community-dwelling older adults in The Netherlands. The Dutch National Food Consumption Survey (DNFCS) and the Healthy Life in an Urban Setting study (HELIUS) are important sources of data on dietary intake and nutritional status in The Netherlands. However, for adults aged 65 years and older, the available datasets are small or partially outdated. The DNFCS 2019–2021 [6] includes a nationally representative sample (*n* = 3570, aged 1–79 years), of whom 607 were aged 65–79 years. Results for this group are available only as one of the standard age–sex strata in the general reporting. Adults aged 80 years and older were not included at all. A previous DNFCS conducted in 2010–2012 [7] focused specifically on adults aged 70 years and older (*n* = 739), yet these data are more than a decade old. The HELIUS study (2011–2013) [8] provides dietary data for several ethnic groups in Amsterdam (*n* = 4200, aged 18–70 years). Approximately 1800 participants were aged 51–70 years, but intakes are reported by ethnicity and sex only, not by age group. Consequently, the number of participants aged 65 years and older cannot be determined from published reports. Moreover, HELIUS did not collect data on adults above the age of 70, leaving the oldest age groups unrepresented.

Quantitative data on dietary intake and diet quality capture only part of the picture. Equally important is how older adults themselves experience and perceive nutrition and mealtimes. These experiences shape food choice, meal patterns, and the willingness to act on dietary advice, and are therefore a key determinant of whether nutritional guidance translates into behavioural change. A quantitative assessment may reveal that intake of a given nutrient or food group falls below recommended levels, but cannot clarify which individual, social, or environmental factors underlie this shortfall. Understanding the personal, interpersonal, and environmental context in which older adults make daily food decisions is indispensable for developing interventions that genuinely resonate with this population. Qualitative insight into mealtime experiences and nutritional perceptions is thus needed alongside quantitative intake data.

Taken together, a systematic synthesis of both quantitative evidence on dietary intake and diet quality and qualitative evidence on nutritional experiences and mealtime perceptions is needed to clarify what is currently known, identify knowledge gaps, and substantiate strategies aimed at supporting healthy ageing in place.

The aim of this systematic review is therefore to synthesise the available evidence on nutrition among community-dwelling older adults (≥65 years) in The Netherlands, with a focus on (1) dietary intake and diet quality and (2) nutritional experiences and mealtime perceptions.

## 2. Materials and Methods

This systematic review was conducted and reported in accordance with the Preferred Reporting Items for Systematic Reviews and Meta-Analyses (PRISMA 2020) statement [9]. A completed PRISMA 2020 checklist is provided in the Supplementary Materials. The review was not prospectively registered with PROSPERO or another international register, and no separate protocol was prepared.

### 2.1. Eligibility Criteria

In this systematic review, studies were eligible if they reported data on either the dietary intake or diet quality of community-dwelling older adults (≥65 years) in The Netherlands or their experiences and perceptions related to nutrition and mealtimes. Eligible studies were required to report outcomes on macronutrients, micronutrients, energy intake, fluid intake, dietary supplements, or food group consumption, with or without comparison to established dietary guidelines, or qualitative findings reflecting the views or experiences of community-dwelling adults aged ≥65 years. Intake data were also considered when a study reported them only as a participant characteristic or covariate.

Eligible articles were full-text publications in Dutch or English that reported data collected from January 2015 onward to ensure relevance to current dietary patterns, healthcare context, and ageing population.

All studies meeting the predefined eligibility criteria were considered, regardless of participant demographics. No restrictions were applied related to cultural background, ethnicity, or socioeconomic status. This approach ensured that the review captured the full range of available evidence on dietary intake, diet quality, and experiences and perceptions on nutrition and mealtimes in older adults in The Netherlands.

### 2.2. Search Strategy

The search was structured with the SPIDER framework [10], which was developed as an alternative to PICO for qualitative and mixed-methods evidence synthesis and therefore fits a review combining both types of evidence. Because SPIDER has been reported to favour specificity over sensitivity and may miss relevant records [10], the search strings were kept deliberately broad and complemented by forward and backward citation searching. The framework guided both the construction of the search strings and the formulation of the eligibility criteria. PubMed, Scopus, and EBSCOhost/CINAHL were searched on 29 September 2026 for quantitative, qualitative, and mixed-methods studies. The searches were limited to publications from 2015 onward in English or Dutch. The PubMed search strategy was developed by B.v.d.W. and subsequently reviewed and optimised by H.S.B. and an information specialist from The Hague University of Applied Sciences. Key topics were “elderly”, “independent living”, “dietary intake”, “eating experience” and “Dutch population”. The full PubMed search strategy is provided in the Supplementary Materials (Table S6). The strategies for Scopus and EBSCOhost/CINAHL were derived from this strategy by translating search terms into a database-specific syntax.

In addition to the above-mentioned databases, the websites of the Nederlands Tijdschrift voor Voeding & Diëtetiek and VoedingNu were searched for relevant information. In addition to the primary search strategy, forward and backward citation searching was performed to expand the search for relevant information and to identify additional sources that met the inclusion criteria.

### 2.3. Selection Process

The articles were imported into Rayyan.ai and duplicates were removed. Two reviewers (B.v.d.W. and H.S.B.) independently screened the records at title and abstract level. Discrepancies were resolved through discussion, using the eligibility criteria as reference. A third reviewer would have been consulted if disagreement had persisted, but this was not required. Full-text reports were likewise assessed independently by both reviewers. Differences at full-text level were resolved through discussion, and all final decisions were reached by consensus. When a report described a substudy of a larger study, the main report of that study was retrieved and assessed as well. Reasons for full-text exclusion were recorded using the eligibility criteria in a fixed order (publication type, population, data collection period, outcome). Two further reasons were applied last: reports on the same participants as an included study without additional intake data, and studies in which participants were selected on low intake or malnutrition, as their intake might not represent the community-dwelling population. After selection, the articles were categorised by topic, corresponding to either dietary intake and diet quality or experiences and perceptions related to nutrition and mealtimes.

### 2.4. Data Collection Process

For each included study, B.v.d.W. extracted relevant data using a standardised data extraction form. No automated tools were used for data extraction. Because single extraction has been shown to generate more errors than double extraction [11], a second reviewer (H.S.B.) independently extracted the same items from the source publications. The two extractions were compared item by item, and differences were resolved through discussion. Where only counts were reported, percentages were calculated from the reported numerators and denominators, and where only one of two complementary categories was reported, the other was calculated from it. These values are marked in the tables of included studies (Section 3). No attempts were made to contact study investigators to obtain or confirm missing or unclear data.

For studies on dietary intake and diet quality, the following items were extracted: author and year of publication, study population (sample size, mean age, sex), data collection method, outcome variables (macronutrients, micronutrients, energy intake (including, where reported, its relation to estimated energy expenditure), fluid intake, dietary supplements, food group consumption), and corresponding reported values. For studies on experiences and perceptions related to nutrition and mealtimes, the following items were extracted: author and year of publication, study population, research method, key themes, and key findings.

For all outcome variables, all reported values were extracted regardless of measurement method or time point. No data were extracted on funding sources or other study-level characteristics beyond those directly relevant to the research questions.

### 2.5. Risk of Bias Assessment

The methodological quality of included studies was assessed independently by two reviewers (B.v.d.W. and A.M.H.H.) using the critical appraisal tools from the Joanna Briggs Institute (JBI) (Supplementary Materials, Tables S1–S5). The checklist corresponding to each study design was applied: the checklists for analytical cross-sectional studies, prevalence studies, quasi-experimental studies, randomised controlled trials, and qualitative research. Each study was evaluated against the checklist criteria, and for every item it was indicated whether the criterion was met (yes), not met (no), insufficiently reported (unclear), or not applicable. Discrepancies between the two reviewers were resolved through discussion.

### 2.6. Synthesis Method

Due to the heterogeneity of study designs, populations, and outcome measures across the included studies, a narrative synthesis was performed. Results of individual studies were tabulated and grouped thematically into dietary intake and diet quality and experiences and perceptions related to nutrition and mealtimes.

No missing summary statistics were imputed. Apart from the values calculated from reported counts or percentages (Section 2.4), data were used as reported in the original studies. No meta-analysis was conducted.

### 2.7. Reporting Bias Assessment

The potential for reporting bias was considered during the synthesis. Given the small number of studies included and the heterogeneity in methods and populations, formal assessment of publication bias was not feasible. Instead, the risk of selective reporting was considered during critical appraisal using the JBI checklists.

## 3. Results

### 3.1. Study Selection

**Figure 1:**
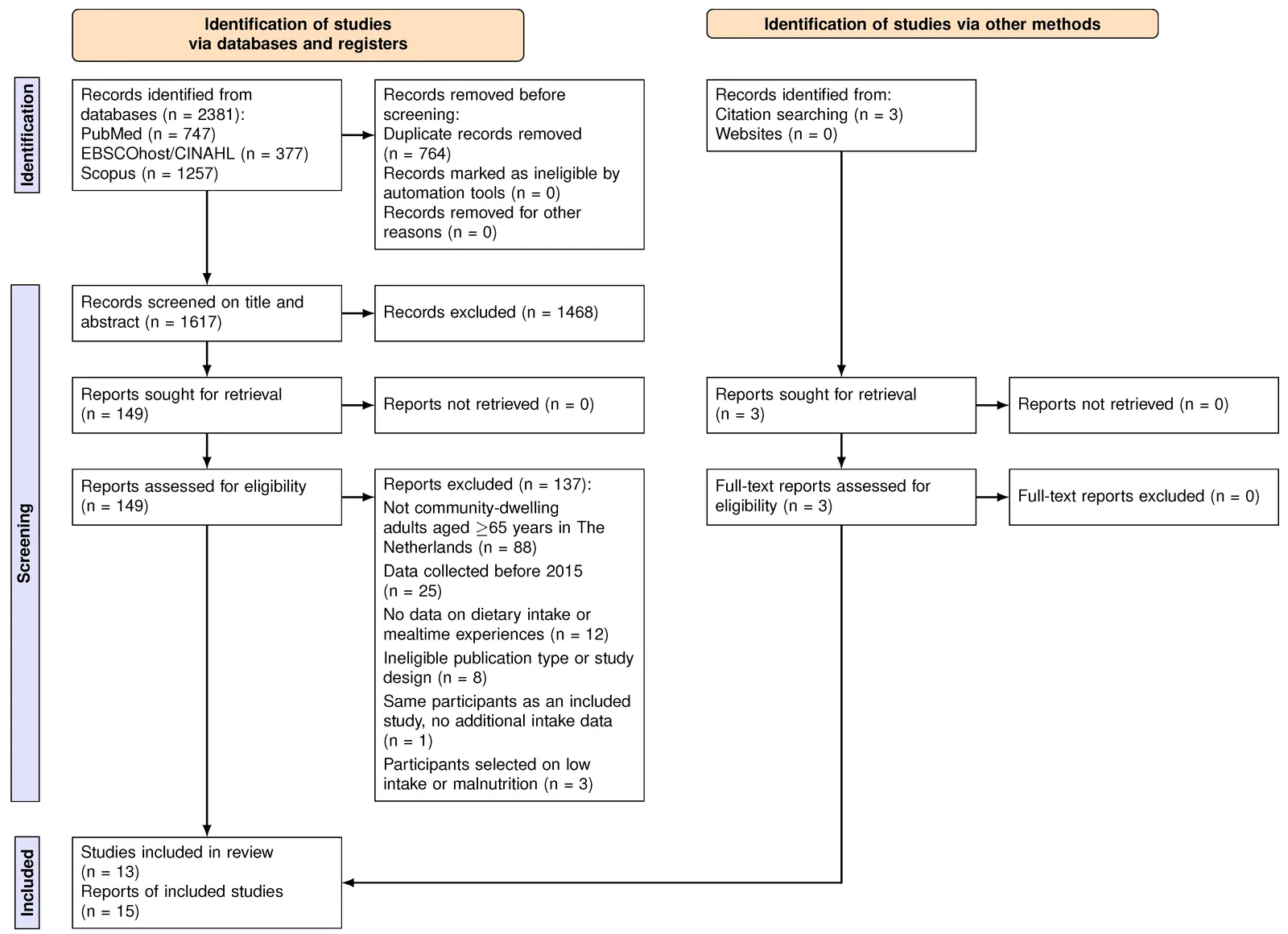
PRISMA 2020 flow diagram of the study selection process. Source: Page et al. [9] (CC BY 4.0).

Figure 1 presents the selection process. A total of 2381 records were retrieved from the databases, 0 from the websites of the Nederlands Tijdschrift voor Voeding & Diëtetiek and VoedingNu, and 3 additional reports through forward and backward citation searching. After removal of duplicates, 1617 records were screened on title and abstract, of which 149 were sought for retrieval. Together with the 3 reports from citation searching, 152 reports were assessed for eligibility. Of the 149 database records, 137 were excluded. The reasons are given in Figure 1, and each excluded report is listed with its reason in Table S7. The most frequent reasons were a population that was not community-dwelling, not aged 65 years and older, or not Dutch (*n* = 88) and data collected before 2015 (*n* = 25). One report concerned the same participants as an included study without additional intake data, and three studies were excluded because their participants had been selected on a low intake or malnutrition, so that their data did not describe a general population. All 3 reports identified through citation searching were included. Fifteen reports, describing thirteen independent study populations, met the inclusion criteria [12,13,14,15,16,17,18,19,20,21,22,23,24,25,26]. Study #11 reports on a subset (≥70 years, *n* = 94) of the participants of the ConsuMEER trial (#10), and study #12 analyses a subgroup of the DNFCS 2019–2021 sample on which study #9 is based. Thirteen publications examined dietary intake or diet quality (Table 1) [12,13,14,15,16,17,18,19,20,21,22,23,24], and two studies investigated experiences related to nutrition and mealtimes (Table 2) [25,26]. Throughout the Results, individual studies are referred to by the numbers assigned to them in Table 1 and Table 2 (#1–#15).

### 3.2. Study Characteristics

The results show a heterogeneous set of outcomes based on a variety of research methods. The thirteen included articles (Table 1) show outcomes of energy intake and expenditure, protein consumption, vitamin D intake and supplement use, alcohol usage, and consumption of food products of community-dwelling people aged 65 years and older. In addition, specific cut-off values and the Dutch Healthy Diet-index Food Frequency Questionnaire (DHD-FFQ) provide insight into diet quality. Study #11 [22] is a substudy of the ConsuMEER trial (study #10 [21]) and reports on a subset (≥70 years, *n* = 94) of its participants. Study #12 [23] analyses the 70–79-year-old subgroup of the DNFCS 2019–2021, the survey on which study #9 [20] is based. The findings from the included studies on these outcomes are presented in Section 3.4.

Nutritional status, in terms of (risk of) malnutrition, was not an outcome of this review, but several studies reported it as a characteristic of their study population, which helps to place the intake findings in context. In study #1, 68.2% of the Amsterdam subcohort had a normal nutritional status according to the Mini Nutritional Assessment (MNA) and 31.8% were at risk of malnutrition. Studies #4 and #8 also used the MNA: in study #4, about one in five participants were at risk of undernutrition, and in study #8 the mean score indicated a normal nutritional status. Study #7 reported the prevalence of undernutrition risk with an adapted version of the ${\mathrm{SNAQ}}^{65+}$ in a second online sample, and study #10 assessed undernutrition risk with the ${\mathrm{SNAQ}}^{65+}$ and SCREEN II at baseline. The remaining studies did not report a validated measure of nutritional status.

Two qualitative studies met the inclusion criteria (Table 2). Study #14 [25] conducted fifteen semi-structured interviews and participant observations among community-dwelling older adults (*n* = 15, mean age 76.9 ± 6.4 years, 73% female), focusing on interpersonal determinants of eating behaviour. Study #15 [26] employed a multi-method qualitative design combining focus groups (*n* = 17), photovoice (*n* = 30), and two separate interview studies (*n* = 20 and *n* = 40). Both studies focused on Dutch community-dwelling adults aged 65 years and older living in The Netherlands.

### 3.3. Risk of Bias in Included Studies

The methodological quality of the thirteen quantitative studies was appraised with the JBI critical appraisal checklists appropriate to each design (Supplementary Materials, Tables S1–S4): the checklist for analytical cross-sectional studies for #1, #2, #3, #5, #6, #9, #11, #12 and #13, for prevalence studies for #7, which reported the prevalence and determinants of undernutrition in a self-selected sample recruited through online self-tests, for quasi-experimental studies for #4 and #8, and for randomised controlled trials for #10. Studies were considered of adequate quality when they met the majority of applicable criteria. Eight of the nine cross-sectional studies met this threshold and #11 met half of the criteria, although none met every criterion. The most frequent weaknesses were the use of non-validated or single-item self-report measures of exposure or outcome (#2, #3, #6) or of an exposure derived post hoc from a questionnaire not designed for it (#5), a non-standard criterion for the condition (#3, #9) and the absence of confounder control (#1, #11, #12). Study #7, appraised as a prevalence study, showed the weakest profile (five items “No”), reflecting a self-selected online sample without a defined sampling frame or response rate and an unvalidated modification of the ${\mathrm{SNAQ}}^{65+}$. The quasi-experimental studies were limited by non-randomised allocation with baseline imbalance, differences between the groups in other care and differential attrition (#4), and by the absence of a control group, 45% attrition and a change in questionnaire administration mode between measurements (#8). The RCT (#10) was adequately randomised but showed baseline imbalance in protein intake, incomplete blinding and randomisation of couples as a unit without corresponding analysis, so its effect estimate is likely to be less precise than reported. Study #12 shows the same pattern as the other survey-based studies: crude subgroup comparisons without confounder control, and supplement doses derived from label information. Study #13 used validated scales and controlled for confounders, but relied on a convenience sample with a high proportion of higher-educated and digitally reachable older adults, and its regression table contained reporting inconsistencies. Overall, the risk of bias was moderate. Findings from #4, #7, #8 and #11 should be interpreted with greater caution.

Critical appraisal of the two qualitative studies (#14, #15) with the JBI checklist for qualitative research showed a clear difference between them (Table S5). Study #14 met eight of ten criteria: no philosophical perspective was stated (Q1) and the influence of the researchers on the research was only partly addressed (Q7), but the report does include a statement locating the authors professionally (Q6), a detailed account of the analysis and ample representation of participants’ voices. Study #15 met five criteria and was rated unclear on three (Q4, Q5, Q7) because the analysis of two of its four sub-studies was only briefly described, findings were partly presented as counts and the influence of the researchers was addressed only procedurally. In addition, no philosophical perspective was stated (Q1) and the researchers were not located culturally or theoretically (Q6). The findings of study #15 should therefore be interpreted with more caution.

### 3.4. Dietary Intake and Diet Quality

#### 3.4.1. Energy and Protein Intake

In study #5, mean energy intake was 2264 kcal/day in men and 1934 kcal/day in women in a physically active sample. In study #1, energy intake was more than 10% below estimated total energy expenditure (energy deficit) in 68.2% of the Amsterdam subcohort. Protein consumption showed a comparable trend. Study #1 reported both an energy and protein deficit in 13 out of 22 participants (59.1%), based on a <1.2 g/kg BW cut-off, and study #6 reported a high chance of low protein intake in almost 40% of 824 Dutch community-dwelling older adults, based on a <1.0 g/kg BW cut-off. In study #10, mean protein intake at baseline was 1.09 and 0.99 g/kg BW/day in the two groups, and 27–32% of participants reached 1.2 g/kg BW/day. DHD-FFQ protein subscores ranged from moderate (4.9 and 5.3 at baseline in study #4) to low (2.2 at baseline in study #8). Although these studies used different cut-offs, assessment methods and populations, and their estimates are therefore not directly comparable, together they indicate that a substantial proportion of participants did not meet protein intake recommendations.

#### 3.4.2. Vitamin D Intake

Across three studies (#4, #5, #8), dietary vitamin D intake appeared uniformly low. Dietary intake was consistently far below the Dutch recommendation (10 µg/day up to 69 years and 20 µg/day from 70 years), with a mean intake of 4.0 µg/day in study #5, where 99% of participants had an intake below 20 µg/day. These figures concern dietary intake, not vitamin D status: study #5, the only study that also measured serum 25-hydroxyvitamin D, found a mean summertime concentration of 88.8 nmol/L in physically active older adults who did not use supplements, with only 2% below 50 nmol/L. DHD-FFQ vitamin D subscores confirmed this picture (1.8 in study #8 and 2.2–2.5 in study #4, on a 0–10 scale), pointing to a limited intake of vitamin D-rich foods. Vitamin D supplement use was assessed in two studies (#11, #12). Study #11 found that 41.5% of participants used no vitamin D supplement at all, and only 51.1% supplemented in line with the Dutch guideline (≥20 µg/day). The proportion using any vitamin D supplement (58.5%) was nevertheless higher than the 23% reported for the national survey of older adults the authors used as reference. The nationally representative DNFCS 2019–2021 subsample aged 70–79 years (#12) confirms this picture: half of the participants reported any use of a vitamin D-containing supplement and 40% used one on the recall days, and among users only 35% took exactly the recommended 20 µg/day while 37% took less. Women reported supplement use more often than men (61% vs. 41%).

#### 3.4.3. Consumption of Food Products

Across four studies (#2, #4, #7, #8), consumption patterns varied across food groups and depended strongly on the assessment method used. Fruit intake appeared relatively adequate, with 63.0% of participants meeting the fruit standard in study #2 and consistently high DHD-FFQ fruit subscores in studies #4 (8.2–8.7) and #8 (8.2). The findings for vegetables were less consistent: 62.8% met the vegetable standard in study #2 and DHD-FFQ vegetable subscores ranged from 5.7 (study #8) to 7.2 (study #4), while study #7 (using a stricter cut-off of ≥4 portions of fruit or vegetables per day) found that only 32.5% of participants met this threshold. Intake of other food groups was generally low: in study #7, 55.4% of participants consumed meat, eggs, fish or meat alternatives once a day or less, 55.3% consumed dairy products once a day or less, and 24.9% reported ≤4 glasses of fluid per day. DHD-FFQ fish subscores in studies #4 (5.3–5.4) and #8 (5.6) were similarly moderate.

Across these food groups, fruit intake was relatively high, whereas the findings for vegetables were mixed and the intake of other product categories was frequently below recommended levels.

#### 3.4.4. Overall Diet Quality Using DHD-FFQ

Two studies (#4 and #8) assessed overall diet quality using the DHD-FFQ, which evaluates adherence to Dutch dietary guidelines across eight domains on a scale from 0 to 80. Total scores were 57.3 and 56.5 (#4) and 62.0 (#8), indicating reasonable but suboptimal dietary adherence.

### 3.5. Experiences and Mealtime Perceptions

Despite differences in primary focus and design, the two studies yielded largely convergent findings. The results were synthesised narratively around four themes: knowledge and awareness, attitudes and resistance to change, barriers, and promoters.

#### 3.5.1. Knowledge and Awareness

Study #15 showed that older adults have limited awareness of the nutritional importance of protein intake. Participants associated healthy eating primarily with fruit and vegetable consumption, while awareness of protein needs was low. Nearly half of the interview participants (19 of 40) reported little to no knowledge of protein, none could indicate the recommended daily intake, and a substantial proportion had an inaccurate self-assessment of their own consumption when compared with the Protein Screener 55+. Study #14 did not assess protein knowledge specifically, but reported that older adults framed their eating behaviour primarily in terms of individual factors such as habits, taste, and health, alongside environmental factors such as food availability, with interpersonal considerations not arising spontaneously. Although the two studies approach the topic from different angles, both suggest that protein intake is not a salient concern in this population.

#### 3.5.2. Attitudes and Resistance to Change

Both studies documented strong attachment to existing dietary patterns. Participants described their eating behaviour as habitual and satisfactory, rooted in lifelong traditions and personal preferences. In study #15, almost all photovoice participants (98%) indicated they were unwilling to alter their diet, and some focus-group and interview participants explicitly dismissed the relevance of increased protein intake at their age. Participants in study #15 also considered themselves sufficiently skilled and knowledgeable to compose a healthy diet and saw no need for protein-enriched products. Study #14 similarly reported that eating patterns were perceived as fixed and that changes in social circumstances such as widowhood or divorce reduced the effort participants were willing to invest in meal preparation. Together, these findings point to dietary habits that are strongly ingrained and resistant to change.

#### 3.5.3. Barriers

The identified barriers spanned individual, interpersonal, and environmental domains. At the individual level, study #15 identified lack of knowledge and resistance to changing habits as central obstacles, alongside physical and mental deterioration, including reduced mobility affecting grocery shopping. Study #14 did not formulate explicit barriers, but described habitual eating as a deeply ingrained pattern rooted in upbringing and tradition, and found that living alone reduced the perceived effort worth investing in meal preparation and diminished the enjoyment of eating. Study #14 further identified a strong social norm whereby older adults do not comment on each other’s eating behaviour, which may limit opportunities for positive social influence. At the environmental level, study #15 reported that constantly changing dietary recommendations and an overwhelming product range in supermarkets created confusion, while product-related issues such as difficult packaging and, for some participants, small portion sizes posed additional challenges.

#### 3.5.4. Promoters

Several factors were identified that could support improved dietary behaviour. Study #15 found that older adults place considerable trust in healthcare professionals. Study #15 additionally identified product offers as a motivator for trying unfamiliar foods, and found that television, radio, and regional newspapers were the preferred information channels. Study #14 highlighted organised social eating activities in the neighbourhood as a valued opportunity for social contact, particularly for those living alone, and noted that informal exchanges between older adults could inspire dietary experimentation. The neighbourhood-specific context of study #14, characterised by accessible local shops and services, partly explained why participants made limited use of social support for grocery shopping, preferring to maintain their autonomy.

## 4. Discussion

This systematic review aimed to gain insight into the dietary intake, diet quality, and mealtime experiences of community-dwelling older adults aged 65 years and older in The Netherlands. Fifteen publications, reporting on thirteen independent study populations, were included: thirteen examining dietary intake or diet quality (of which #11 reports on a subset of the trial participants of #10, and #12 on a subgroup of the survey sample of #9), and two exploring experiences and perceptions related to nutrition and mealtimes.

The review confirms that suboptimal nutrition among community-dwelling Dutch older adults is a real and persistent problem. Across the included studies, three findings recur consistently: a substantial proportion of participants does not meet protein recommendations (although the studies differ in cut-off, assessment method and population), dietary vitamin D intake is consistently below the recommended level, and the consumption of fish, dairy, and meat (alternatives) is suboptimal, vegetable intake is mixed, and fruit intake is relatively adequate. The qualitative evidence adds that deeply ingrained habits, limited awareness of protein needs, and reluctance to change form important underlying mechanisms. Together, these findings suggest potential targets for prevention of malnutrition and may indicate a more structural nutritional vulnerability in this population. Given the variation in study quality and measurement approaches, these findings should be interpreted as indicative patterns rather than precise prevalence estimates.

### 4.1. Findings in the Context of Existing Evidence

At a higher level of abstraction, the picture emerging from this review is broadly in line with both Dutch and international evidence. Within The Netherlands, the vitamin D shortfall aligns with the DNFCS 70+ data (2010–2012), in which protein intake was sufficient for the majority but lower among older adults with functional limitations, dairy consumption was less than half the recommended amount and at least 40% did not eat oily fish weekly [7]. A low consumption of dairy and of meat, fish and eggs was seen several years later in the largest included sample, drawn from the online screening tools of the Dutch Malnutrition Steering Group (#7) [18], suggesting that at least the low dairy intake has persisted for about a decade. The modelling study on DNFCS 2019–2021 data included in this review (#9) [20] indicates that protein adequacy in current diets is largely maintained in flexitarian, pescetarian and vegetarian scenarios, but becomes inadequate for most older adults in a fully vegan scenario. Internationally, declining appetite and energy intake with advancing age [27], a high prevalence of protein intake below the recommended 1.0 g/kg body weight per day [28] across cohorts from Europe and North America [29], and widespread vitamin D deficiency [30,31] are all consistent with what the included Dutch studies show. It should be noted, however, that the included studies mainly assessed dietary vitamin D intake. Only study #5 also measured serum 25-hydroxyvitamin D and found a high summertime status in physically active older adults who did not use supplements, despite a low dietary intake. Dietary intake alone is therefore not equivalent to vitamin D status, and this review does not establish the prevalence of vitamin D deficiency in this population.

The qualitative themes identified here also recur in international qualitative work. Habitual eating and resistance to dietary change are reported in Swedish, Austrian and British studies [32,33,34] and in an international meta-synthesis [35]. Limited nutritional knowledge is described in Austrian, British and Chinese studies [33,34,36] and in the meta-synthesis [35], and trust in healthcare professionals as a source of nutritional information in Chinese work [36]. The Austrian study [33] examined older adults in need of care and their informal caregivers, which limits direct comparison with the present population. The Dutch evidence does not deviate from the international picture but rather adds a national layer to a recognisable pattern. The interpersonal observation that older adults tend not to comment on each other’s eating behaviour represents a culturally specific feature with direct relevance for intervention design, but does not contradict the broader European literature.

Interpreted through the DoMAP model, these findings map onto several determinants of malnutrition. Low protein and vitamin D intake reflect the “low intake” mechanism, while the qualitative themes (ingrained habits, limited nutritional awareness, living alone, and a social norm of non-interference) correspond to psychological, social, and behavioural determinants. Viewing the results in this way underlines that dietary intake in this population is shaped by interacting determinants rather than a single cause, consistent with the framework introduced at the outset.

The specifically Dutch contribution of this review therefore lies less in the direction of the findings than in situating them within the Dutch dietary guidelines, the DHD-FFQ, and local care structures.

The included studies also offer some clues as to why protein and vitamin D intake in particular fall short. These clues come from associations that several studies examined alongside intake. They were not among the outcomes extracted for this review and rest on a small number of studies. For protein, study #6 [17] found that lower scores on all behavioural determinants studied were associated with a higher chance of low protein intake, and that knowledge and social support remained independently associated when all determinants were modelled together. The low awareness of protein needs reported in study #15 [26] points in the same direction. Appetite forms a second route: in study #7 [18], participants aged 85 years and older scored worst on almost all nutritional risk factors, including appetite and meal preparation, and study #3 [14] linked poorer appetite to lower mood, poorer sleep and, in men, more severe pain. Study #9 [20] adds that protein adequacy in current Dutch diets depends largely on animal-based foods, which makes it sensitive to a shift towards fully plant-based eating. For vitamin D, the explanation appears to lie mainly in food choice: fish, one of the few foods that contribute substantially to vitamin D intake, was consumed below recommendations by most participants in study #2 [13] and received moderate DHD-FFQ subscores in studies #4 and #8. In study #5 [16], dietary vitamin D intake was not associated with serum 25-hydroxyvitamin D, whereas outdoor physical activity was, which underlines that intake and status reflect different processes. The relative adequacy of fruit intake, finally, may reflect that fruit is easy to consume and culturally familiar, whereas adequate protein intake depends more on portion size, cost, and appetite.

Sociodemographic factors were examined only sporadically. In study #6, sociodemographic differences between respondents with a high and a low chance of low protein intake were small, and the associations with behavioural determinants were attenuated but largely persisted after adjustment for age, sex, BMI, living situation and income. In study #3, the association between low mood and poor appetite was stronger in women. Study #2 concerned older adults with a Surinamese background, almost half of whom had a low income, and in study #14 [25] eating habits were described as rooted in upbringing and tradition. A systematic analysis of how socioeconomic position, household composition, cultural background, generation and sex shape intake in this population was not possible with the available evidence.

Evidence on the relation between diet quality and socio-psychological factors was similarly limited, as none of the included studies related an overall diet quality score to such factors. Several findings nevertheless suggest that this relation matters. In study #2, meeting the guidelines for fruit and vegetables was positively associated with well-being, as was frequently visiting family and friends. Social support was independently associated with a lower chance of low protein intake in study #6, and in study #14 living alone reduced both the effort participants were willing to invest in meal preparation and the enjoyment of eating, while organised social eating was valued. Low mood, in turn, was associated with poorer appetite in study #3. Within the DoMAP model, these factors correspond to the psychological and social determinants that act on intake. As these associations come from observational, mostly cross-sectional studies, the direction of these relations cannot be established.

### 4.2. Limitations of Review Method and Included Studies

Full-text screening, data extraction and critical appraisal were each performed by two reviewers (Section 2.3, Section 2.4 and Section 2.5), which limits the risk of selection and extraction errors.

The decision to exclude studies with data collected prior to 1 January 2015 was a deliberate methodological choice, based on the reasoning that population dietary patterns change over time, as shown for Dutch adults aged 19–69 years between the DNFCS 2007–2010 and 2012–2016 surveys [37], and that older data are therefore less likely to reflect the current older adult population. While this enhanced the contemporary relevance of the evidence base, it may also have excluded potentially valuable earlier studies, resulting in a narrower synthesis than a broader time frame would have allowed.

Given the substantial heterogeneity in study designs, populations, and outcome measures, a meta-analysis was not feasible and a narrative synthesis was performed instead. This approach, while appropriate given the diversity of included studies, does not allow for pooled effect estimates or formal statistical comparison across studies. Conclusions are therefore inherently more descriptive.

Finally, no restrictions were applied with regard to cultural background, ethnicity, or socioeconomic position. While this inclusive approach ensured comprehensive evidence capture, it also means that culturally specific dietary patterns and food-related experiences could not be evaluated separately.

Several limitations related to the included studies should also be considered. The thirteen quantitative studies differed substantially in dietary assessment method, including FFQs, multi-day food diaries, 24-h recalls, screening tools, and questionnaires or interviews on supplement use, which complicates direct comparison of findings. Sample sizes varied considerably, with the smallest study including only 20 participants, of whom 11 completed the study, limiting statistical reliability. Some studies recruited self-selected participants, for instance through online self-tests (#7), which introduces selection bias. Study populations were highly diverse, ranging from physically active older adults to geriatric outpatients, home care clients, and older adults with a Surinamese background, meaning findings cannot be straightforwardly generalised to all community-dwelling older adults in The Netherlands. The pilot study #8 and the trial #4 recruited home care clients in the same region in successive years. Whether some older adults took part in both could not be determined from the reports, and the two studies were treated as independent study populations. Taken together with the risk-of-bias assessment, these limitations mean that the strength of evidence varies across outcomes: findings on protein and vitamin D, which recur across multiple studies, are more robust than those resting on single or methodologically weaker studies.

The qualitative evidence base comprises only two studies, one of which (#15) has substantial methodological limitations (Supplementary Materials, Table S5), which is insufficient to draw robust or generalisable conclusions. The themes identified should be regarded as exploratory rather than definitive, and as a starting point for further qualitative research rather than a comprehensive picture. These limitations should be kept in mind when interpreting the practical implications discussed below.

### 4.3. Implications for Practice

Three findings from the included studies are directly relevant to practice. First, protein and dietary vitamin D intake fell short of recommendations across several studies, and fish, dairy and meat (alternatives) were frequently consumed below recommended levels. Second, such shortfalls were also observed in populations that were largely well nourished according to screening tools: in study #1, 59.1% of a subcohort in which 68.2% had a normal MNA score had both an energy and a protein deficit, and in study #4, in which about one in five participants were at risk of undernutrition, DHD-FFQ protein and vitamin D subscores were moderate to low. Screening for nutritional status alone may therefore not identify all older adults with inadequate intake. Third, the two qualitative studies suggest that dietary habits are strongly ingrained, that awareness of protein needs is low, and that older adults place considerable trust in healthcare professionals. As these findings rest on two studies, one of which has substantial methodological limitations, they should be regarded as exploratory.

The following implications are derived from these findings but were not evaluated in this review. They should be read as suggestions for practice rather than established effects. For dietitians, the findings support a more proactive, preventive role in the community, in which attention to protein and vitamin D intake is not reserved for visibly underweight or clinically referred older adults, and in which advice builds on existing meal patterns through small, incremental adjustments rather than radical change. General practitioners and practice nurses (*POH-Ouderen*) could include brief questions on protein-rich foods, vitamin D supplementation and meal patterns in regular consultations, with a low-threshold referral to a dietitian where needed. District and home care nurses may pick up cues in the home environment, such as reduced mobility affecting grocery shopping or simplified meal preparation in older adults living alone. Beyond healthcare, the qualitative studies tentatively point to organised community meals and traditional media (television, radio, regional newspapers) as possible routes to reach this group.

Taken together, these suggestions indicate that improving the nutritional situation of community-dwelling older adults is not a task for dietitians alone and may require a multidisciplinary approach.

### 4.4. Directions for Future Research

Several knowledge gaps emerge from this synthesis and point directly to priorities for future research. First, there is a clear need for a contemporary, nationally representative analysis of dietary intake and diet quality among Dutch adults aged 65 years and older: the most recent dedicated survey of older adults (DNFCS 2010–2012, aged ≥70 years) is more than a decade old, and the DNFCS 2019–2021 covers adults up to 79 years only, without a dedicated analysis of the overall diet of older adults. Such an analysis could build on the existing DNFCS 2019–2021 data for adults aged 65–79 years, stratified by age band, sex, socioeconomic status, ethnicity, household composition, and frailty status, and should be extended to adults aged 80 years and older. Second, the considerable heterogeneity in dietary assessment methods across the included studies highlights the need for greater methodological standardisation, so that findings can be meaningfully compared and synthesised. Third, the most pronounced gap concerns qualitative evidence: only two qualitative studies were available, which is insufficient to capture the personal, social, and environmental factors that shape daily food practices among Dutch older adults. Further qualitative work is needed, including studies that specifically address older adults living alone, older adults with a non-Dutch cultural background, and older adults who are shifting towards more plant-based dietary patterns. Fourth, intervention studies are needed that evaluate the effectiveness of habit-sensitive, incremental dietary strategies that build on existing meal patterns and trusted information channels, rather than requiring radical dietary change. Finally, given the ongoing protein transition, more research is needed into how a shift towards plant-based patterns can be implemented safely in older adults without compromising protein adequacy. Simulation work suggests that the risk of inadequate protein intake and amino acid quality becomes most pronounced under full plant-based scenarios and depends strongly on the choice of plant-based substitutes [38], while qualitative work in Dutch adults aged 55–74 indicates mixed attitudes towards such a transition, with health considerations, taste, and habits as recurring drivers, and a consistent need for enhanced knowledge and practical tools to make such a shift independently [39]. However, the current evidence base in older adults is limited, and much of the available evidence is indirect or model-based rather than derived from real-world dietary behaviour.

## 5. Conclusions

Overall, the conclusions of this review should be interpreted as a synthesis of heterogeneous and partly low-sample evidence, rather than as precise estimates of dietary adequacy in the Dutch older population. This systematic review suggests that the dietary intake of community-dwelling older adults in The Netherlands is suboptimal in several key areas, most notably protein, dietary vitamin D, and various food groups including dairy, fish, and meat alternatives, while fruit consumption appears relatively adequate and vegetable intake is mixed. Where overall diet quality was assessed with the DHD-FFQ, scores indicated reasonable but suboptimal adherence to Dutch dietary guidelines. The limited qualitative evidence suggests that deeply ingrained habits, low nutritional awareness, and reluctance to change are important underlying factors, yet the scarcity of qualitative studies represents a significant knowledge gap. Targeted, habit-sensitive nutritional guidance and further research, particularly into how older adults themselves experience and perceive food and mealtimes, are needed to support effective strategies for healthy ageing in place.

## Figures and Tables

**Table 1:** Dietary intake and diet quality characteristics of included studies in community-dwelling older adults.

**Study 1** **Reference:** Yeung, 2020 [12] **Population:** Community-dwelling older adults referred to a geriatric outpatient mobility clinic (Amsterdam) for falls and/or poor balance; *n* = 22; median age 81.2 y; 63.6% female ^b^ (Melbourne cohort excluded). **Assessment method:** QUARK indirect calorimetry (RMR); 3-day food diary (2 weekdays + 1 weekend day).	
**Variable**	**Result**
Energy intake (kcal/d)	1851 (407)
ΔTEE−intake (kcal/d)	402 (445)
Energy balance, *n* (%)	5 (22.7)
Energy deficit, *n* (%)	15 (68.2)
Energy excess, *n* (%)	2 (9.0)
Protein intake (g/d), median [IQR]	70.3 [60.0–82.3]
Protein intake (g/kg BW), median [IQR]	0.99 [0.87–1.20]
Protein deficit <1.2 g/kg BW, *n* (%)	17 (77.3)
Energy + protein deficit, *n* (%)	13 (59.1)
Energy deficit only, *n* (%)	2 (9.1)
Protein deficit only, *n* (%)	4 (18.2)
No deficit, *n* (%)	1 (4.5)
**Study 2** **Reference:** Jagroep, 2022 [13] **Population:** Community-dwelling older adults (≥70 y) of Surinamese background in Rotterdam; *n* = 697 (reported as 679 in the methods section of the source); mean age 76.2 (4.9) y; 54.2% female. **Assessment method:** Self-administered postal questionnaire.	
**Variable**	**Result**
Fish, % meeting standard	40.9
Fruit, % meeting standard	63.0
Vegetables, % meeting standard	62.8
**Study 3** **Reference:** Wijnhoven, 2024 [14] **Population:** Community-dwelling older adults ≥75 y (Longitudinal Aging Study Amsterdam, LASA); included sample *n* = 434 (M 80.9 (4.9), F 81.5 (4.9) y; 58.8% female ^b^); excluded sample *n* = 252 (M 84.7 (5.4), F 85.6 (6.2) y; 61.1% female ^b^). **Assessment method:** LASA 2015–2016 wave; self-reported average number of alcoholic drinks per day (none; ≤2/day; >2/day), available for 170 M/242 F (included) and 39 M/55 F (excluded). Shown as male/female.	
**Variable**	**Result**
None, included, M/F (%)	14.7/25.2
≤2 drinks/d, included, M/F (%)	53.5/63.6
>2 drinks/d, included, M/F (%)	31.8/11.2
None, excluded, M/F (%)	23.1/40.0
≤2 drinks/d, excluded, M/F (%)	66.7/52.7
>2 drinks/d, excluded, M/F (%)	10.3/7.3
**Study 4** **Reference:** Van Doorn-van Atten, 2018 [15] **Population:** Community-dwelling older adults ≥65 y receiving home care and/or living in sheltered accommodation (about one in five at risk of undernutrition); intervention *n* = 97 (78.4 y; 66.0% female ^b^), control *n* = 107 (81.0 y; 76.6% female ^b^); baseline (T0). Values shown as intervention/control. **Assessment method:** DHD-FFQ.	
**Variable**	**Result**
Total DHD-FFQ (0–80)	57.3 (10.0)/56.5 (10.2)
Vegetables (0–10)	6.3 (2.7)/7.2 (2.7)
Fruit (0–10)	8.2 (2.4)/8.7 (2.5)
Dietary fibre (0–10)	7.4 (1.8)/8.1 (1.8)
Fish (0–10)	5.4 (3.2)/5.3 (3.4)
TFA, % compliant	83.2/70.2
SFA (0–10)	5.0 (4.3)/4.5 (4.0)
Sodium (0–10)	7.0 (2.6)/6.3 (2.7)
Alcohol (0–10)	9.5 (1.4)/9.3 (2.1)
Protein (0–10) ^a^	4.9 (3.6)/5.3 (3.6)
Vitamin D (0–10) ^a^	2.5 (1.5)/2.2 (1.1)
**Study 5** **Reference:** Ten Haaf, 2019 [16] **Population:** Physically active, Caucasian older adults ≥65 y not using vitamin D supplements; *n* = 450; mean age 71.9 y; 22% female ^b^. **Assessment method:** Online FFQ (180 items, 1-month reference); two 24-h recalls for participants unable to complete the online FFQ (*n* = 30).	
**Variable**	**Result**
Energy intake (kcal/d), M/F	2264 (650)/1934 (463)
Vitamin D from diet (µg/d)	4.0 (1.9)
Alcohol (g/d)	14.4 (14.6)
Alcohol: non-drinker, *n* (%)	99 (22)
Alcohol: low, *n* (%)	289 (64)
Alcohol: moderate, *n* (%)	45 (10)
Alcohol: high, *n* (%)	7 (2)
**Study 6** **Reference:** Verwijs, 2022 [17] **Population:** Community-dwelling older adults ≥65 y; *n* = 824; mean age 72.9 y; 62.5% female. **Assessment method:** Protein Screener 55+ (Pro55+; risk score, cut-off 0.3).	
**Variable**	**Result**
Low chance of low protein intake (<1.0 g/kg BW/d), *n* (%)	499 (60.6)
High chance of low protein intake, *n* (%)	325 (39.4)
**Study 7** **Reference:** Borkent, 2019a [18] **Population:** Community-dwelling older adults ≥65 y; *n* = 2470; mean age 74.3 y; 75.5% female. **Assessment method:** SCREEN II (Seniors in the Community: Risk Evaluation for Eating and Nutrition, version II).	
**Variable**	**Result**
Fruit/vegetable ≥ 4/d, *n* (%)	802 ^b^ (32.5)
Fruit/vegetable ≤ 3/d, *n* (%)	1668 (67.5)
Meat/eggs/fish or substitute ≤ 1/d, *n* (%)	1369 (55.4)
Meat/eggs/fish or substitute > 1/d, *n* (%)	1101 ^b^ (44.6)
Dairy ≤ 1/d, *n* (%)	1367 (55.3)
Dairy > 1/d, *n* (%)	1103 ^b^ (44.7)
Fluid ≤ 4 glasses/d, *n* (%)	616 (24.9)
Fluid ≥ 5 glasses/d, *n* (%)	1854 ^b^ (75.1)
**Study 8** **Reference:** Van Doorn-van Atten, 2019 [19] **Population:** Dutch home care clients ≥65 y; *n* = 20 (mean age 80.6 y; 75% female); DHD-FFQ values at baseline (M0) for the 11 completers. **Assessment method:** DHD-FFQ.	
**Variable**	**Result**
Total DHD-FFQ (0–80)	62.0 (5.9)
Vegetables (0–10)	5.7 (1.8)
Fruit (0–10)	8.2 (2.3)
Dietary fibre (0–10)	6.8 (2.0)
Fish (0–10)	5.6 (2.8)
SFA (0–10)	8.0 (3.0)
Trans fatty acids (0–10)	10.0 (0.0)
Sodium (0–10), median [IQR]	8.5 [0.7]
Alcohol (0–10)	9.6 (1.5)
Protein (0–10) ^a^	2.2 (2.3)
Vitamin D (0–10) ^a^	1.8 (0.6)
**Study 9** **Reference:** Borkent, 2024 [20] **Population:** Community-dwelling older adults aged 65–79 y from the DNFCS 2019–2021; *n* = 607; median age M 71, F 69 y; 49% female. Values shown as male/female. **Assessment method:** Two non-consecutive 24-h dietary recalls.	
**Variable**	**Result**
Energy (kcal/d), median [IQR]	2188 [1947–2455]/1741 [1539–1956]
Plant-based protein (% of total)	39.0/37.7
Protein intake (g/d), median	82.5/67.9
Protein intake (g/kg BW/d), median	0.96/0.94
Utilizable protein (g/kg BW/d), median	0.85/0.82
<EAR (<0.58 g/kg/d) utilizable protein (%)	7.5/11.1
**Study 10** **Reference:** Borkent, 2019b [21] **Population:** Older adults ≥65 y; *n* = 98; mean age 80.4 y; 61% female; baseline. Values shown as intervention/control. **Assessment method:** 3-day food diary.	
**Variable**	**Result**
Total energy (kcal/d)	2037 (SE 78)/1871 (SE 76)
Protein, breakfast (g)	13.4 (SE 1.0)/14.1 (SE 0.9)
Protein, lunch (g)	18.2 (SE 1.1)/15.9 (SE 1.2)
Protein, dinner (g)	38.7 (SE 2.5)/33.7 (SE 2.2)
Total daily protein (g)	80.4 (SE 4.1)/73.9 (SE 3.3)
Protein (g/kg BW/d)	1.09 (SE 0.05)/0.99 (SE 0.05)
≥1.2 g/kg BW/d (%)	31.9/26.5
**Study 11** **Reference:** Beelen, 2017 [22] **Population:** Older adults ≥70 y; *n* = 94; mean age 80.8 y; 61.7% female. **Assessment method:** Interviewer-administered supplement inventory (brand and dose) at a home visit, verified against packaging.	
**Variable**	**Result**
Vitamin D supplement sufficient (≥20 µg/d), %	51.1
Vitamin D supplement insufficient (<20 µg/d), %	7.4
No vitamin D supplement, %	41.5
**Study 12** **Reference:** Verbakel, 2026 [23] **Population:** Community-dwelling adults aged 70–79 y from the DNFCS 2019–2021 (nationally representative sample; subgroup of the same survey as study #9); *n* = 331; 54% male; 45% lower educated. Values shown as general questionnaire/24-h recall (average of two recall days), weighted for socio-demographic factors, season and day of the week. **Assessment method:** General questionnaire on frequency of use of vitamin D-containing supplements; two non-consecutive 24-h dietary recalls (GloboDiet) with supplement brand, quantity and dose, linked to the Dutch Supplement Database.	
**Variable**	**Result**
Use of a vitamin D-containing supplement (%)	50/40
Supplement use, women/men (general questionnaire, %)	61/41
Vitamin D-only supplement among users (%)	56/54
Multivitamin-mineral supplement among users (%)	24/35
(Almost) daily use among users of a vitamin D-only supplement (general questionnaire, %)	86
Supplement dose below/exactly/above the recommended 20 µg/d (24-h recall, % of users)	37/35/28
**Study 13** **Reference:** Huisman, 2026 [24] **Population:** Community-dwelling adults ≥65 y in the Northern Netherlands (Groningen, Friesland, Drenthe), recruited April–July 2024 through purposeful and convenience sampling; *n* = 216; 57% female; 57% aged 65–74 y, 40% 75–84 y, 4% ≥85 y; 58% higher educated. **Assessment method:** Self-administered questionnaire (paper or digital), including alcohol consumption (glasses/week).	
**Variable**	**Result**
Alcohol (glasses/week; *n* = 210), median [IQR]	2 [0–6]

Values are mean (SD) unless otherwise specified. Study #11 reports on a subset of the participants of the ConsuMEER trial (#10), and study #12 analyses a subgroup (70–79 years) of the DNFCS 2019–2021 sample on which study #9 is based; studies #1–#13 therefore represent eleven independent study populations. ^a^ Additional scores for protein and vitamin D added by the study authors; not part of the DHD-FFQ total (0–80). ^b^ Calculated from the reported counts or percentages (as the complement of the reported category, or from the reported percentage of the other sex). Abbreviations: BW, body weight; DHD-FFQ, Dutch Healthy Diet-index Food Frequency Questionnaire; DNFCS, Dutch National Food Consumption Survey; EAR, Estimated Average Requirement; F, female; FFQ, Food Frequency Questionnaire; IQR, interquartile range; M, male; RMR, resting metabolic rate; SE, standard error; SFA, saturated fatty acid; TEE, total energy expenditure; TFA, trans fatty acid.

**Table 2:** Study characteristics of qualitative studies on eating and meal experience in older adults (studies #14 and #15, continuing the numbering from Table 1).

**Study 14** **Reference:** Bukman, 2020 [25] **Population:** Independently living older adults ≥65 y in one Rotterdam neighbourhood, recruited with the intention of selecting persons at increased risk of falling (not verified); *n* = 15; mean age 76.9 (6.4) y; 73% female ^a^. **Method:** Semi-structured interviews and participant observations at three shopping locations (5 observations) and three collective eating activities (5 observations).	
**Interview topics**	**Results: Eating/Meal Experience**
Food choice motivesTraditionsHabitsDefinition of a proper mealSkipping mealsEating alone/with othersGrocery shoppingMeal preparationFormal meal supportSocial tiesLonelinessChanges in diet	Older adults did not directly identify interpersonal factors as influences on eating behaviour, instead pointing to individual factors (e.g., habits, health status) or environmental factors (e.g., food accessibility). Interpersonal factors did, however, appear to influence eating behaviour. Four key interpersonal themes: (1) behaviour is shaped by one’s context, (2) living alone influences (determinants of) eating behaviour in multiple ways, (3) a salient norm exists that people do not interfere with others’ eating behaviour, and (4) limited use of social support (formal and informal) for grocery shopping and cooking, except for organised neighbourhood eating activities.
**Study 15** **Reference:** Linschooten, 2021 [26] **Population:** Community-dwelling older adults ≥65 y; focus groups *n* = 17 (mean age 80.4 y; 12 F, 5 M); photovoice *n* = 30 (mean age 72.0 y; 60% female); interview study I *n* = 20 (mean age 75.3 y; 70% female); interview study II *n* = 40 (mean age 78.4 y; 57.5% female). **Method:** Four qualitative studies: focus groups (*n* = 17), photovoice (*n* = 30), interview study I (*n* = 20), interview study II (*n* = 40).	
**Interview topics**	**Results: Eating/Meal Experience**
Healthy eatingAttitude towards high-protein productsHabitual dietary behaviourGrocery shoppingMeal preparationConsumption patternHealthy ageingKnowledge/influenceCommunication channelsDietary protein knowledgeCommunication preferences	Community-dwelling older adults showed low awareness and urgency regarding adequate protein intake, possibly linked to low knowledge levels. After being informed of daily protein requirements, 17 of 40 participants had no accurate indication of their own intake: 11 overestimated and 6 underestimated.

^a^ Percentage calculated from the reported counts. Abbreviations: F, female; M, male.

## Data Availability

The data extraction form template and the extracted data from included studies are available from the corresponding author on request. No analytic code was generated, as no statistical synthesis was performed. The full search strategy is reported in the Supplementary Materials (Table S6). The list of included studies and JBI appraisal results are presented in Table 1 and Table 2 and in the Supplementary Materials.

## Supplementary Materials

The following supporting information can be downloaded at: https://www.mdpi.com/article/10.3390/nu18213590/s1, Table S1: JBI critical appraisal of analytical cross-sectional studies; Table S2: JBI critical appraisal of prevalence studies; Table S3: JBI critical appraisal of quasi-experimental studies; Table S4: JBI critical appraisal of randomised controlled trials; Table S5: JBI critical appraisal of qualitative research; Table S6: Full PubMed search strategy; Table S7: Reports excluded at full-text assessment, with the reason for exclusion; File S1: PRISMA 2020 checklist.

## Abbreviations

The following abbreviations are used in this manuscript:

DHD-FFQ	Dutch Healthy Diet-index Food Frequency Questionnaire
DNFCS	Dutch National Food Consumption Survey
DoMAP	Determinants of Malnutrition in Aged Persons
EAR	Estimated Average Requirement
FFQ	Food Frequency Questionnaire
HELIUS	Healthy Life in an Urban Setting
JBI	Joanna Briggs Institute
LASA	Longitudinal Aging Study Amsterdam
MNA	Mini Nutritional Assessment
PRISMA	Preferred Reporting Items for Systematic Reviews and Meta-Analyses
RCT	Randomised Controlled Trial
SCREEN II	Seniors in the Community: Risk Evaluation for Eating and Nutrition, version II
${\mathrm{SNAQ}}^{65+}$	Short Nutritional Assessment Questionnaire 65+
SPIDER	Sample, Phenomenon of Interest, Design, Evaluation, Research type
