# Supplementary material for: Dietary Intake, Diet Quality, and Mealtime Experiences of Community-Dwelling Older Adults in The Netherlands: A Systematic Review
Source: Nutrients. 2026 Oct 29;18(21):3590. doi: 10.3390/nu18213590 (PMC13765346; doi:10.3390/nu18213590)
Supplement: Supplementary file 1 [file nutrients-18-03590-s001.zip › Supplementary Materials S1-S7.pdf]

Supplementary Materials

Dietary Intake, Diet Quality, and Mealtime Experiences of Community-Dwelling Older Adults in The Netherlands: A Systematic Review

Bart van der Wijst, Henriëtte S. Brummer, Rosalie van der Vaart, Astrid M.H. Horstman, Luc P. de Witte

This file presents the Joanna Briggs Institute (JBI) critical appraisal results for each included study, grouped by study design (Tables S1–S5), the full PubMed search strategy (Table S6), and the reports excluded at full-text assessment with the reason for each exclusion (Table S7). In all appraisal tables, Q = checklist question, Y = criterion met, N = criterion not met, U = unclear or insufficiently reported, NA = not applicable. Study numbers correspond to Tables 1 and 2 of the main article. The most recent versions of the JBI critical appraisal tools were used, with the questions in their original order: the tools for the assessment of risk of bias for analytical cross-sectional studies (2026, Barker et al., JBI Evid Synth 2026;24(3):401–8), quasi-experimental studies (2023, Barker et al., JBI Evid Synth 2024;22(3):378–88) and randomised controlled trials (2023, Barker et al., JBI Evid Synth 2023;21(3):494–506), the checklist for qualitative research (2024) and the checklist for prevalence studies (2020). Study #7 is appraised as a prevalence study. For the other study types the classification follows Tables 1 and 2.

Table S1: JBI critical appraisal of analytical cross-sectional studies.

#	Q1	Q2	Q3	Q4	Q5	Q6	Q7	Q8
1	Y	Y	Y	Y	N	N	Y	Y
2	Y	Y	U	Y	Y	Y	Y	Y
3	Y	N	U	U	Y	Y	Y	Y
5	Y	Y	U	Y	Y	Y	Y	Y
6	Y	Y	U	Y	Y	Y	Y	Y
9	Y	N	Y	U	NA	NA	Y	Y
11	Y	Y	N	Y	N	N	U	Y
12	Y	Y	Y	Y[a]	N	N	Y	Y
13	Y	Y	Y[b]	Y	Y	Y	U	Y

Q1 Inclusion criteria clearly defined. Q2 Objective, standard criteria for measurement of the condition. Q3 Exposure measured validly/reliably. Q4 Outcomes measured validly/reliably. Q5 Confounders identified. Q6 Strategies to deal with confounders stated. Q7 Appropriate statistical analysis. Q8 Subjects and setting described in detail (comprehensiveness of reporting, not a risk-of-bias item). Study #3: baseline data of a longitudinal diary study. Study #9: modelling study on national survey data, confounding items not applicable to within-person simulation. [a] Study #12: use of a vitamin D-containing supplement measured validly (two 24-h recalls and a questionnaire). Supplement dose unclear (label-based doses, inexact product matching). [b] Study #13: social network, education and income measured validly. Health literacy unclear (three-item screener originally developed for clinical patients, with a ceiling effect). Q7 rated U because of reporting inconsistencies in the regression table.

Table S2: JBI critical appraisal of prevalence studies.

#	Q1	Q2	Q3	Q4	Q5	Q6	Q7	Q8	Q9
7	N	N	Y	N	U	N	U	Y	N

Q1 Sample frame appropriate. Q2 Appropriate sampling/recruitment. Q3 Adequate sample size. Q4 Subjects/setting described in detail. Q5 Sufficient coverage in analysis. Q6 Valid methods to identify the condition. Q7 Condition measured in a standard, reliable way. Q8 Appropriate statistical analysis. Q9 Response rate adequate/managed. Q6 rated N because the adjusted SNAQ^{65+} (BMI replacing arm circumference) is described by the authors as not validated. SCREEN II is validated. Q7 rated U because the condition was measured in a standard way with SCREEN II but not with the adjusted SNAQ^{65+}.

Table S3: JBI critical appraisal of quasi-experimental studies.

#	Q1	Q2	Q3	Q4	Q5	Q6	Q7	Q8	Q9
4	Y	Y	N	N	Y	Y	Y	U	Y
8	Y	N	NA	NA	N	U	U	N	Y

Q1 Cause and effect clear. Q2 Control group present. Q3 Groups comparable. Q4 Similar treatment (other than intervention). Q5 Multiple pre/post measurements. Q6 Outcomes measured in the same way. Q7 Outcomes measured reliably. Q8 Follow-up complete/described. Q9 Appropriate statistical analysis. #4: Q4 rated N because the groups differed in other care, such as following a diet and receiving nursing care, which may affect the outcomes. Q8 rated U because attrition was differential (22% vs 6%) and drop-outs were described but not analysed by group. #8: single-arm design, 9 of 20 participants dropped out and only completers were analysed.

Table S4: JBI critical appraisal of randomised controlled trials.

#	Q1	Q2	Q3	Q4	Q5	Q6	Q7	Q8	Q9	Q10	Q11	Q12	Q13
10	Y	Y	N	Y	N	Y	N	Y	Y	Y	Y	Y	N

Q1 True randomisation. Q2 Allocation concealment. Q3 Groups similar at baseline. Q4 Participants blinded. Q5 Those delivering treatment blinded. Q6 Groups treated identically other than the intervention. Q7 Outcome assessors blinded. Q8 Outcomes measured in the same way. Q9 Outcomes measured reliably. Q10 Follow-up complete/differences described and analysed. Q11 Analysed in the groups to which they were randomised. Q12 Appropriate statistical analysis. Q13 Trial design appropriate and deviations accounted for. Q3 N: baseline imbalance including in protein intake. Q13 N: couples randomised as a unit without corresponding analysis, two participants allocated non-randomly.

Table S5: JBI critical appraisal of qualitative research.

#	Q1	Q2	Q3	Q4	Q5	Q6	Q7	Q8	Q9	Q10
14	N	Y	Y	Y	Y	Y	U	Y	Y	Y
15	N	Y	Y	U	U	N	U	Y	Y	Y

Q1 Congruity philosophy–methodology. Q2 Congruity methodology–research question. Q3 Congruity methodology–data collection. Q4 Congruity methodology–analysis. Q5 Congruity methodology–interpretation. Q6 Researcher positioning stated. Q7 Reflexivity addressed. Q8 Participants/voices represented. Q9 Ethics/ethical approval. Q10 Conclusions flow from analysis. #14: Q6 rated Y on the basis of the “Authors’ information” section. #15: Q4 and Q5 rated U because the analysis of two of the four sub-studies is only briefly described and findings are partly presented as counts. Q7 rated U because the influence of the researchers is addressed only procedurally.

Table S6: Full PubMed search strategy. The strategies for Scopus and EBSCOhost/CINAHL were derived from this strategy by translating the search terms into a database-specific syntax.

Searched 29 September 2026. Limits: publication date from 5 June 2015, English or Dutch, humans.
(“Retirement”[MeSH Terms] OR “Aged”[MeSH Terms] OR “elder*”[Text Word] OR “eldest”[Text Word] OR “frail*”[Text Word] OR “geriatri*”[Text Word] OR “old”[Text Word] OR “senior*”[Text Word] OR “senium”[Text Word] OR “septuagenarian*”[Text Word] OR “octagenarian*”[Text Word] OR “octogenarian*”[Text Word] OR “nonagenarian*”[Text Word] OR “centarian*”[Text Word] OR “centenarian*”[Text Word] OR “supercentenarian*”[Text Word] OR “community-dwelling”[Text Word] OR “ageing”[Text Word] OR “aging”[Text Word] OR “older”[Text Word] OR “ouderen”[Text Word] OR “bejaard*”[Text Word] OR “retire*”[Text Word]) **AND** (“Independent Living”[MeSH Terms] OR “Geriatric Assessment”[MeSH Terms] OR “indepen*”[Text Word] OR “autonom*”[Text Word] OR “self relian*”[Text Word] OR “self sufficien*”[Text Word] OR “living at home”[Text Word] OR “homebound”[Text Word] OR “home-based”[Text Word] OR “homestay*”[Text Word] OR “community-dwelling”[Text Word] OR “home-dwelling”[Text Word] OR “non-institutionalized”[Text Word] OR “aging in place”[Text Word] OR “living alone”[Text Word] OR “home living old* adults”[Text Word] OR “elder* at home”[Text Word]) **AND** ((“diet, food, and nutrition”[MeSH Terms] OR “Feeding Behavior”[MeSH Terms] OR “Nutrition Therapy”[MeSH Terms] OR “Drinking Behavior”[MeSH Terms] OR “Health Status”[MeSH Terms] OR “Malnutrition”[MeSH Terms] OR “Protein-Energy Malnutrition”[MeSH Terms] OR “eating*”[Text Word] OR “drink*”[Text Word] OR “nutr*”[Text Word] OR “diet*”[Text Word] OR “food intake”[Text Word] OR “food behavior”[Text Word] OR “Feeding Behavior”[Text Word] OR “appetite”[Text Word] OR “Malnutrition”[Text Word] OR “undernutrition”[Text Word] OR “fluid intake”[Text Word] OR “hydration”[Text Word] OR “water intake”[Text Word] OR “fluid balance”[Text Word] OR “dehydration”[Text Word] OR “fluid consumption”[Text Word] OR “water consumption”[Text Word] OR “thirst”[Text Word] OR “Nutrition Assessment”[MeSH Terms] OR “Nutrition Surveys”[MeSH Terms] OR “Diet Records”[MeSH Terms]) OR (“health knowledge, attitudes, practice”[MeSH Terms] OR “Patient Compliance”[MeSH Terms] OR “Food Insecurity”[MeSH Terms] OR “Food Preferences”[MeSH Terms] OR “Social Environment”[MeSH Terms] OR “Access to Healthy Foods”[MeSH Terms] OR “Psychology”[MeSH Terms] OR “Social Support”[MeSH Terms] OR “Psychosocial Support Systems”[MeSH Terms] OR “Healthy Aging”[MeSH Terms] OR “barriers”[Text Word] OR “challenges”[Text Word] OR “difficult*”[Text Word] OR “food access”[Text Word] OR “Food Insecurity”[Text Word] OR “positive experience*”[Text Word] OR “health attitude*”[Text Word] OR “health knowledge”[Text Word] OR “health practice*”[Text Word] OR “Social Support”[Text Word] OR “food preference*”[Text Word] OR “food habit*”[Text Word] OR “Healthy Aging”[Text Word] OR “experiences with health”[Text Word] OR “food perception”[Text Word] OR “food experience”[Text Word] OR “meal experience”[Text Word] OR “food-related emotions”[Text Word])) **AND** (“Dutch people”[Supplementary Concept] OR “Netherlands”[MeSH Terms])

Table S7: Reports excluded at full-text assessment, with the reason for exclusion (PRISMA 2020 item 16b). Reports are grouped by reason, in the order of Figure 1 of the main article, and listed alphabetically within each group. Each report is listed under the first eligibility criterion it did not meet, with the criteria applied in a fixed order: publication type or study design, population, data collection period, outcome. The criteria on overlapping participants and on selection by intake or nutritional status were applied last.

No.	First author	Year	Journal	DOI
**Not community-dwelling adults aged ≥65 years in The Netherlands** (n = 88)				
1	Alferink LJ	2019	Gut	10.1136/gutjnl-2017-315940
2	Alferink LJM	2017	J Hepatol	10.1016/j.jhep.2017.03.013
3	Amankour F	2025	Eur J Ageing	10.1007/s10433-025-00859-9
4	Bagchus C	2015	Health Expect	10.1111/hex.12188
5	Beers S	2026	Br J Nutr	10.1017/S000711452510603X
6	Bleijenberg N	2016	Worldviews Evid Based Nurs	10.1111/wvn.12151
7	Boxum SD	2026	BMC Health Serv Res	10.1186/s12913-026-14047-7
8	Broese van Groenou M	2019	J Fam Issues	10.1177/0192513X19846179
9	Buckland G	2015	Int J Cancer	10.1002/ijc.29411
10	Campos-Obando N	2017	J Bone Miner Res	10.1002/jbmr.3094
11	Chilunga FP	2021	Lancet Healthy Longev	10.1016/S2666-7568(21)00087-8
12	Colyer-Patel K	2026	Alcohol Clin Exp Res (Hoboken)	10.1111/acer.70232
13	Conkova N	2020	Gerontologist	10.1093/geront/gnz125
14	de Groot AJ	2025	Rehabil Nurs	10.1097/RNJ.0000000000000489
15	de Jonge EA	2017	Am J Clin Nutr	10.3945/ajcn.116.139196
16	de Jonge EAL	2017	Osteoporos Int	10.1007/s00198-017-4037-9
17	de Kroon MLA	2021	Nutrients	10.3390/nu13010214
18	Dekker LH	2015	Food Nutr Res	10.3402/fnr.v59.26317
19	Dijkstra SC	2015	Public Health Nutr	10.1017/S1368980014001487
20	Doornenbal BM	2022	Health Soc Care Community	10.1111/hsc.13649
21	Dorhout BG	2025	JMIR Form Res	10.2196/54392
22	Dreyer RP	2014	J Am Heart Assoc	10.1161/JAHA.114.000863
23	Fiorini L	2021	Disabil Rehabil Assist Technol	10.1080/17483107.2019.1642392
24	Fluitman KS	2019	J Nutr Health Aging	10.1007/s12603-019-1241-7
25	Franse CB	2018	Int J Nurs Stud	10.1016/j.ijnurstu.2018.09.006
26	Gharbaran P	2026	BMJ Open	10.1136/bmjopen-2025-108216
27	Govaerts J	2026	Exp Gerontol	10.1016/j.exger.2026.113171
28	Grasso AC	2021	J Nutr	10.1093/jn/nxaa322
29	Hackert MQN	2019	Soc Sci Med	10.1016/j.socscimed.2019.112535
30	Harbers MC	2021	Nutr J	10.1186/s12937-021-00713-5
31	Hartman YAW	2024	Gynecol Oncol	10.1016/j.ygyno.2024.03.007
32	Heath AK	2020	Breast Cancer Res	10.1186/s13058-019-1244-7
33	Heijboer AC	2015	Clin Endocrinol (Oxf)	10.1111/cen.12711
34	Hengeveld LM	2020	J Cachexia Sarcopenia Muscle	10.1002/jcsm.12580
35	Huijgen E	2026	J Nutr	10.1016/j.tjnut.2025.101266
36	Huisman MG	2016	Eur J Surg Oncol	10.1016/j.ejso.2016.03.005
37	Jaarsma E	2024	J Prev Alzheimers Dis	10.14283/jpad.2023.64
38	Jongeneelen ME	2025	BMC Public Health	10.1186/s12889-025-21458-4
39	Karimi L	2016	J Hypertens	10.1097/HJH.0000000000000861
40	Klaassens M	2015	J Aging Stud	10.1016/j.jaging.2015.05.002
41	Leonard SE	2026	BMC Med	10.1186/s12916-026-04941-6
42	Leontjevas R	2016	J Clin Epidemiol	10.1016/j.jclinepi.2016.07.009
43	Loogman L	2021	Dis Colon Rectum	10.1097/DCR.0000000000001976
44	Machado-Fragua MD	2020	Eur J Nutr	10.1007/s00394-019-01984-9
45	Meesters PD	2019	Community Ment Health J	10.1007/s10597-019-00389-6
46	Melo van Lent D	2017	Br J Nutr	10.1017/S0007114517000319
47	Mendonca N	2021	Am J Clin Nutr	10.1093/ajcn/nqab051
48	Merritt MA	2016	Am J Clin Nutr	10.3945/ajcn.115.118588
49	Mertens E	2019	Nutr J	10.1186/s12937-018-0425-z
50	Mutlu U	2016	Medicine (Baltimore)	10.1097/MD.0000000000005477
51	Nijholt W	2016	J Nutr Health Aging	10.1007/s12603-015-0610-0
52	Niskanen RT	2023	J Hum Nutr Diet	10.1111/jhn.13197
53	Niskanen RT	2025	Eur Geriatr Med	10.1007/s41999-025-01267-z
54	Paans NPG	2017	Psychiatry Res	10.1016/j.psychres.2017.04.044
55	Paans NPG	2019	Appetite	10.1016/j.appet.2018.12.030

continued on next page

Table S7 (continued)

No.	First author	Year	Journal	DOI
56	Papadimitriou N	2020	Eur J Nutr	10.1007/s00394-019-02132-z
57	Peetoom KKB	2016	Technol Disabil	10.3233/TAD-160441
58	Prevo L	2018	Int J Qual Stud Health Well-being	10.1080/17482631.2018.1542909
59	Ramsey KA	2022	Clin Interv Aging	10.2147/CIA.S353573
60	Reinders I	2022	Eur J Nutr	10.1007/s00394-021-02675-0
61	Rinkel RN	2016	Eur Arch Otorhinolaryngol	10.1007/s00405-015-3680-z
62	Rood PP	2016	Eur J Emerg Med	10.1097/MEJ.0000000000000186
63	Schrier E	2016	Qual Life Res	10.1007/s11136-015-1060-1
64	Smeets SM	2024	BMJ Open	10.1136/bmjopen-2024-088069
65	Tsai LT	2023	Geriatr Gerontol Int	10.1111/ggi.14588
66	van de Rest O	2016	Aging (Albany NY)	10.18632/aging.100877
67	van den Boom W	2023	Public Health Rep	10.1177/00333549231176000
68	van den Helder J	2020	J Cachexia Sarcopenia Muscle	10.1002/jcsm.12634
69	van der Avoort CMT	2021	J Nutr Health Aging	10.1007/s12603-020-1520-3
70	van der Klei VMGTh	2025	Age Ageing	10.1093/ageing/afaf022
71	van der Linden M	2025	Eur J Nutr	10.1007/s00394-025-03683-0
72	van Deudekom FJ	2019	Head Neck	10.1002/hed.25714
73	van Loon SLM	2020	Obes Surg	10.1007/s11695-019-04244-1
74	van Orten-Luiten AC	2016	Eur J Clin Pharmacol	10.1007/s00228-016-2016-2
75	Vergroesen JE	2023	Eur J Nutr	10.1007/s00394-022-03003-w
76	Verlaan S	2017	J Frailty Aging	10.14283/jfa.2017.23
77	Verlinden VJ	2016	Eur J Clin Nutr	10.1038/ejcn.2015.120
78	Verwijs MH	2020	Health Expect	10.1111/hex.13075
79	Viana Veloso GG	2016	Cancer	10.1002/cncr.29862
80	Viramontes Horner D	2020	J Hum Nutr Diet	10.1111/jhn.12764
81	Visser M	2020	Nutrients	10.3390/nu12123708
82	Visser M	2020	Nutrients	10.3390/nu12071998
83	Visser M	2021	Nutrients	10.3390/nu13031006
84	Westland GJ	2015	J Ren Nutr	10.1053/j.jrn.2014.10.004
85	Wijnhoven HAH	2025	Clin Nutr Open Sci	10.1016/j.nutos.2025.05.008
86	Winkens LHH	2023	Health Place	10.1016/j.healthplace.2023.103054
87	Wu J	2026	Nat Commun	10.1038/s41467-026-68791-9
88	Yeung SSY	2020	Arch Gerontol Geriatr	10.1016/j.archger.2020.104066
**Data collected before 2015** (n = 25)				
89	Brinkman S	2018	Arch Gerontol Geriatr	10.1016/j.archger.2018.02.006
90	Brouwer-Brolsma EM	2015	J Am Med Dir Assoc	10.1016/j.jamda.2015.02.013
91	Brouwer-Brolsma EM	2015	Nutrients	10.3390/nu7095364
92	Brouwer-Brolsma EM	2016	Eur J Nutr	10.1007/s00394-015-0970-6
93	de Breij S	2021	Gerontology	10.1159/000512049
94	de Goeij LC	2020	Nutrients	10.3390/nu12020468
95	de Koning EJ	2017	J Psychosom Res	10.1016/j.jpsychores.2017.04.005
96	Douma L	2017	Gerontologist	10.1093/geront/gnv119
97	El Hilali J	2016	J Steroid Biochem Mol Biol	10.1016/j.jsbmb.2015.12.001
98	Gobbens RJJ	2021	Qual Life Res	10.1007/s11136-021-02790-9
99	Hamers JP	2016	J Am Geriatr Soc	10.1111/jgs.13946
100	Ling CHY	2021	Aging Clin Exp Res	10.1007/s40520-020-01639-4
101	Lourida I	2021	Nutrients	10.3390/nu13082574
102	Mangen MJ	2017	Health Qual Life Outcomes	10.1186/s12955-016-0577-5
103	Peek ST	2016	Gerontology	10.1159/000430949
104	Schaap LA	2018	Clin Nutr	10.1016/j.clnu.2017.11.004
105	Scheufele P	2024	Age Ageing	10.1093/ageing/afae040
106	Schoufour JD	2019	Br J Nutr	10.1017/S0007114518003367
107	van der Pols-Vijlbrief R	2017	J Nutr Health Aging	10.1007/s12603-017-0872-9
108	van Dijk SC	2016	Vasc Med	10.1177/1358863X15622281
109	van Houwelingen AH	2015	PLoS One	10.1371/journal.pone.0121013
110	van Lieshout MRJ	2018	J Nutr Health Aging	10.1007/s12603-018-1101-x
111	van Rijn M	2016	Age Ageing	10.1093/ageing/afw157
112	van Seben R	2019	Clin Rehabil	10.1177/0269215518791663
113	van Wijngaarden E	2016	BMJ Open	10.1136/bmjopen-2015-009895
**No data on dietary intake or mealtime experiences** (n = 12)				
114	Bunt S	2021	Ageing Soc	10.1017/S0144686X21000830

continued on next page

Table S7 (continued)

No.	First author	Year	Journal	DOI
115	Hollaar V	2025	Int J Dent Hyg	10.1111/idh.12886
116	Hupkens S	2021	Health Soc Care Community	10.1111/hsc.13071
117	Hussein H	2024	Gerontologist	10.1093/geront/gnad154
118	Mertens EJ	2026	Front Aging	10.3389/fragi.2026.1850676
119	Moens IS	2022	BMC Geriatr	10.1186/s12877-021-02737-2
120	op het Veld LPM	2017	BMC Geriatr	10.1186/s12877-017-0583-4
121	Qi Y	2026	Exp Gerontol	10.1016/j.exger.2025.113006
122	Vaes AMM	2019	Eur J Nutr	10.1007/s00394-018-1634-0
123	van Hees S	2017	Health Place	10.1016/j.healthplace.2017.08.007
124	van Loon IN	2019	Nephron	10.1159/000501277
125	van Veen S	2025	BMC Palliat Care	10.1186/s12904-025-01888-y
**Ineligible publication type or study design** (n = 8)				
126	Franse CB	2017	BMC Geriatr	10.1186/s12877-017-0606-1
127	Reinders I	2020	BMJ Open	10.1136/bmjopen-2020-040637
128	Schols JMGA	2019	Ned Tijdschr Tandheelkd	10.5177/ntvt.2019.12.19059
129	van Deudekom FJ	2016	Acta Oncol	10.3109/0284186X.2015.1096022
130	van Dongen EJI	2018	BMC Public Health	10.1186/s12889-018-5788-8
131	van Doorn-van Atten MN	2018	BMC Geriatr	10.1186/s12877-018-0973-2
132	van Loon M	2021	Tijdschr Gerontol Geriatr	10.36613/tgg.1875-6832/2021.03.02
133	Veleva BI	2019	J Am Med Dir Assoc	10.1016/j.jamda.2018.11.001
**Same participants as an included study, no additional intake data** (n = 1)				
134	van Doorn-van Atten MN	2018	Nutrients	10.3390/nu10081062
**Participants selected on low intake or malnutrition** (n = 3)				
135	Behrouzi P	2020	J Nutr	10.1093/jn/nxz269
136	Fleurke M	2026	J Hum Nutr Diet	10.1111/jhn.70290
137	Grasso AC	2022	Eur J Nutr	10.1007/s00394-022-02896-x

Totals: 137 reports, corresponding to the numbers in Figure 1 of the main article.
