# Supplementary material for: Dietary Intake, Diet Quality, and Mealtime Experiences of Community-Dwelling Older Adults in The Netherlands: A Systematic Review
Source: Nutrients. 2026 Oct 29;18(21):3590. doi: 10.3390/nu18213590 (PMC13765346; doi:10.3390/nu18213590)
Supplement: Supplementary file 1 [file nutrients-18-03590-s001.zip › File S1.PRISMA 2020 checklist.pdf]

Supplementary Materials

PRISMA 2020 Checklist

Systematic review: "Dietary Intake, Diet Quality, and Mealtime Experiences of Community-Dwelling Older Adults in the Netherlands: A Systematic Review" (Bart van der Wijst, Henriëtte S. Brummer, Rosalie van der Vaart, Astrid M.H. Horstman, Luc P. de Witte). Location refers to the section of the manuscript where each item is reported. Based on: Page MJ, et al. BMJ 2021;372:n71. doi:10.1136/bmj.n71.

Section and Topic	Item #	Checklist item	Location where item is reported
**Title**			
Title	1	Identify the report as a systematic review.	Title page
**Abstract**			
Abstract	2	See the PRISMA 2020 for Abstracts checklist.	Abstract (structured: Background/Objectives, Methods, Results, Conclusions)
**Introduction**			
Rationale	3	Describe the rationale for the review in the context of existing knowledge.	Section 1 (Introduction)
Objectives	4	Provide an explicit statement of the objective(s) or question(s) the review addresses.	Section 1 (Introduction), aim and focus points
**Methods**			
Eligibility criteria	5	Specify the inclusion and exclusion criteria for the review and how studies were grouped for the syntheses.	Section 2.1
Information sources	6	Specify all databases, registers, websites, organisations, reference lists and other sources searched or consulted to identify studies. Specify the date when each source was last searched or consulted.	Section 2.2; Table S6
Search strategy	7	Present the full search strategies for all databases, registers and websites, including any filters and limits used.	Section 2.2; Supplementary Materials, Table S6 (full PubMed strategy, with search date and limits)
Selection process	8	Specify the methods used to decide whether a study met the inclusion criteria of the review, including how many reviewers screened each record and each report retrieved, whether they worked independently, and if applicable, details of automation tools used in the process.	Section 2.3
Data collection process	9	Specify the methods used to collect data from reports, including how many reviewers collected data from each report, whether they worked independently, any processes for obtaining or confirming data from study investigators, and if applicable, details of automation tools used in the process.	Section 2.4
Data items	10a	List and define all outcomes for which data were sought. Specify whether all results that were compatible with each outcome domain in each study were sought (e.g. for all measures, time points, analyses), and if not, the methods used to decide which results to collect.	Section 2.4
	10b	List and define all other variables for which data were sought (e.g. participant and intervention characteristics, funding sources). Describe any assumptions made about any missing or unclear information.	Section 2.4
Study risk of bias assessment	11	Specify the methods used to assess risk of bias in the included studies, including details of the tool(s) used, how many reviewers assessed each study and whether they worked independently, and if applicable, details of automation tools used in the process.	Section 2.5 (JBI critical appraisal tools)
Effect measures	12	Specify for each outcome the effect measure(s) (e.g. risk ratio, mean difference) used in the synthesis or presentation of results.	Section 2.6 (narrative synthesis; reported values used as in original studies; no pooled effect measures)
Synthesis methods	13a	Describe the processes used to decide which studies were eligible for each synthesis (e.g. tabulating the study intervention characteristics and comparing against the planned groups for each synthesis (item #5)).	Section 2.6

Continued on next page

Section and Topic	Item #	Checklist item	Location where item is reported
	13b	Describe any methods required to prepare the data for presentation or synthesis, such as handling of missing summary statistics, or data conversions.	Sections 2.4 and 2.6 (no imputation; percentages and complementary counts calculated from reported values where only these were given, marked in Tables 1 and 2; otherwise data used as reported)
	13c	Describe any methods used to tabulate or visually display results of individual studies and syntheses.	Section 2.6; Tables 1 and 2
	13d	Describe any methods used to synthesize results and provide a rationale for the choice(s). If meta-analysis was performed, describe the model(s), method(s) to identify the presence and extent of statistical heterogeneity, and software package(s) used.	Section 2.6 (narrative synthesis; no meta-analysis, given heterogeneity)
	13e	Describe any methods used to explore possible causes of heterogeneity among study results (e.g. subgroup analysis, meta-regression).	Not applicable (no meta-analysis performed)
	13f	Describe any sensitivity analyses conducted to assess robustness of the synthesized results.	Not applicable (no meta-analysis performed)
Reporting bias assessment	14	Describe any methods used to assess risk of bias due to missing results in a synthesis (arising from reporting biases).	Section 2.7
Certainty assessment	15	Describe any methods used to assess certainty (or confidence) in the body of evidence for an outcome.	Section 2.5 (methodological quality via JBI); formal GRADE certainty rating not applied (see Section 4.2)
**Results**			
Study selection	16a	Describe the results of the search and selection process, from the number of records identified in the search to the number of studies included in the review, ideally using a flow diagram.	Section 3.1; Figure 1
	16b	Cite studies that might appear to meet the inclusion criteria, but which were excluded, and explain why they were excluded.	Section 3.1; Figure 1; Table S7 (excluded full-text reports with reasons)
Study characteristics	17	Cite each included study and present its characteristics.	Section 3.2; Tables 1 and 2
Risk of bias in studies	18	Present assessments of risk of bias for each included study.	Section 3.3; Supplementary Materials, Tables S1–S5
Results of individual studies	19	For all outcomes, present, for each study: (a) summary statistics for each group (where appropriate) and (b) an effect estimate and its precision (e.g. confidence/credible interval), ideally using structured tables or plots.	Sections 3.4 and 3.5; Tables 1 and 2
Results of syntheses	20a	For each synthesis, briefly summarise the characteristics and risk of bias among contributing studies.	Sections 3.2–3.5
	20b	Present results of all statistical syntheses conducted. If meta-analysis was done, present for each the summary estimate and its precision (e.g. confidence/credible interval) and measures of statistical heterogeneity. If comparing groups, describe the direction of the effect.	Not applicable (narrative synthesis; no statistical synthesis); see Sections 3.4 and 3.5
	20c	Present results of all investigations of possible causes of heterogeneity among study results.	Not applicable
	20d	Present results of all sensitivity analyses conducted to assess the robustness of the synthesized results.	Not applicable
Reporting biases	21	Present assessments of risk of bias due to missing results (arising from reporting biases) for each synthesis assessed.	Sections 2.7 and 4.2
Certainty of evidence	22	Present assessments of certainty (or confidence) in the body of evidence for each outcome assessed.	Sections 3.3 and 4.2
**Discussion**			
Discussion	23a	Provide a general interpretation of the results in the context of other evidence.	Section 4.1
	23b	Discuss any limitations of the evidence included in the review.	Section 4.2
	23c	Discuss any limitations of the review processes used.	Section 4.2
	23d	Discuss implications of the results for practice, policy, and future research.	Sections 4.3 and 4.4

Continued on next page

Section and Topic	Item #	Checklist item	Location where item is reported
**Other Information**			
Registration and protocol	24a	Provide registration information for the review, including register name and registration number, or state that the review was not registered.	Section 2 (Materials and Methods, opening paragraph): review not registered
	24b	Indicate where the review protocol can be accessed, or state that a protocol was not prepared.	Section 2 (Materials and Methods, opening paragraph): no separate protocol prepared
	24c	Describe and explain any amendments to information provided at registration or in the protocol.	Not applicable
Support	25	Describe sources of financial or non-financial support for the review, and the role of the funders or sponsors in the review.	Funding and Acknowledgments statements
Competing interests	26	Declare any competing interests of review authors.	Conflicts of Interest statement
Availability of data, code and other materials	27	Report which of the following are publicly available and where they can be found: template data collection forms; data extracted from included studies; data used for all analyses; analytic code; any other materials used in the review.	Data Availability Statement

Note: Items marked "Not applicable" concern statistical/meta-analytic procedures that were not performed, as this review used a narrative synthesis owing to heterogeneity in study designs, populations, and outcome measures.
